# Can lower-limb exoskeletons support sit-to-stand motions in frail elderly without crutches? A study combining optimal control and motion capture

**DOI:** 10.3389/fnbot.2024.1348029

**Published:** 2024-04-04

**Authors:** Jan C. L. Lau, Katja Mombaur

**Affiliations:** ^1^CERC Human-Centred Robotics and Machine Intelligence, Systems Design Engineering and Mechanical and Mechatronics Engineering, University of Waterloo, Waterloo, ON, Canada; ^2^BioRobotics Lab, Optimization and Biomechanics for Human Centred Robotics, Institute of Anthropomatics and Robotics (IAR), Karlsruhe Institute of Technology (KIT), Karlsruhe, Germany

**Keywords:** optimal control, sit-to-stand, aging, wearable robotics, modeling, simulation, biomechanics, exoskeleton

## Abstract

With the global geriatric population expected to reach 1.5 billion by 2050, different assistive technologies have been developed to tackle age-associated movement impairments. Lower-limb robotic exoskeletons have the potential to support frail older adults while promoting activities of daily living, but the need for crutches may be challenging for this population. Crutches aid safety and stability, but moving in an exoskeleton with them can be unnatural to human movements, and coordination can be difficult. Frail older adults may not have the sufficient arm strength to use them, or prolonged usage can lead to upper limb joint deterioration. The research presented in this paper makes a contribution to a more detailed study of crutch-less exoskeleton use, analyzing in particular the most challenging motion, sit-to-stand (STS). It combines motion capture and optimal control approaches to evaluate and compare the STS dynamics with the TWIN exoskeleton with and without crutches. The results show trajectories that are significantly faster than the exoskeleton's default trajectory, and identify the motor torques needed for full and partial STS assistance. With the TWIN exoskeleton's existing motors being able to support 112 Nm (hips) and 88 Nm (knees) total, assuming an ideal contribution from the device and user, the older adult would need to contribute a total of 8 Nm (hips) and 50 Nm (knees). For TWIN to provide full STS assistance, it would require new motors that can exert at least 121 Nm (hips) and 140 Nm (knees) total. The presented optimal control approaches can be replicated on other exoskeletons to determine the torques required with their mass distributions. Future improvements are discussed and the results presented lay groundwork for eliminating crutches when moving with an exoskeleton.

## 1 Introduction

The global geriatric population in 2019 was 709 million and is predicted to reach 1.5 billion by 2050 (Division, 2019). With age-associated mobility impairments increasing the susceptibility to falls (Vondracek and Linnebur, 2009; Kalyani et al., 2014), there has been growing interest in tackling this space with robotic interventions. Robotic devices that provide external support to the frailer geriatric user include a sit-to-stand (STS) device for wheelchair users (Zhou et al., 2021) and a walker that can actively assist STS and multi-terrain walking (Mahdi et al., 2022). Their ability to deliver more power to support frailer older adults can come at a cost of occupying more space in the environment. Robotic soft exoskeletons or exosuits are wearable devices often actuated via cables and do not have a bulky external frame (Haufe et al., 2020; Ding et al., 2022). EasyWalk by Siyi Intelligent Technology ^1^ and Myosuit by MyoSwiss ^2^ are exosuits that can also support older adults aside from individuals with motor dysfunction. They are designed to be lightweight and do not restrict user's movements, but they may not be able to support the motions of a frailer older adult due to less power delivery. Robotic lower-limb exoskeletons are another type of wearable devices that can assist human motion in the legs. Their external frame is equipped with motors that can deliver more power than exosuits.

While the stronger motors in robotic lower-limb exoskeletons show potential in supporting frail older adults, the ones currently on the market may not be suitable for them. They are primarily designed for individuals with spinal cord injury or neurological conditions, whose technical characteristics and needs are different from those of the geriatric population (Kapsalyamov et al., 2019). Besides the ATALANTE, which is a 75-kg self-balancing exoskeleton that does not require crutches or walkers (Duburcq et al., 2020), stability aids must be used when moving with devices that do not have frontal actuation, such as the TWIN (Vassallo et al., 2020), Indego (Tefertiller et al., 2017), HAL (Tsukahara et al., 2015), EksoNR (Contreras-Vidal et al., 2016), ReWalk (Contreras-Vidal et al., 2016), and WPAL (Kagawa and Uno, 2009). Particularly for the TWIN, its rigid design lacks frontal degree of freedom (DOF) and does not even allow for small amounts of passive movements. Crutches can provide medial-lateral stability, but they are unnatural to the human gait and prolonged usage can induce upper limb joint deterioration (Opila et al., 1987; Martins et al., 2012).

Exoskeletons support STS, but their pre-programmed trajectories can be very slow. For instance, the TWIN exoskeleton's default STS trajectory takes more than 10 s to complete. We recorded kinematic and force plate data of a healthy young adult following TWIN's pre-programmed motion with crutches. Despite being experienced in moving with the TWIN, the participant commented that its motion is non-intuitive and a lot of coordination is required to move with the default trajectory. Another remark on shoulder extension discomfort related to the ready position is also made, yet this shoulder extension is necessary for a proper push-off for the lifting phase, since the device's default trajectory depends on this push-off for a stable and successful STS. Details on this experiment are elaborated in the Methods and Results sections of this paper.

This motion capture experiment, which is one of a few conducted, confirms that the use of crutches heavily relies on strong and healthy upper limbs. In the context of STS, not only does the user need to know how to move with the exoskeleton, crutch coordination is also necessary. With the TWIN's default trajectory completely dependent on the crutches' push-off, it is not safe to train users on how to follow the device's motion without crutches. Instead, a brand new trajectory, which does not consider any external stability aid, should be created.

Various trajectory generation methods have been done and implemented in lower-limb robotic exoskeletons, and the approaches can be offline or online. Offline means the device's motors move according to the pre-calculated trajectories. Polynomial minimum jerk trajectory, fuzzy logic control, and optimal control are a few offline approaches. Polynomial minimum jerk trajectory uses a polynomial function and minimizes the change in acceleration to yield a smooth trajectory between initial and final positions. Fuzzy logic control is designed to resemble human thinking and can be used for determining joint angles. Optimal control generates a trajectory such that an associated cost function is minimized. These offline methods can be combined with motion capture recordings of healthy human walking data. For instance, TWIN and WPAL utilize motion capture recordings in their polynomial minimum trajectory implementation (Kagawa and Uno, 2009; Vassallo et al., 2020), and ALEX-I has a fuzzy logic control implementation incorporating healthy human walking data (Aphiratsakun and Parnichkun, 2009). Online means the device's movements can adapt to the environment in real-time, such as the Guided Trajectory Learning implementation in the ATALANTE exoskeleton (Duburcq et al., 2020) and a reference trajectory adaptation method that is done in the Indego exoskeleton (Shushtari et al., 2022). However, online approaches limit what one can do, since the trajectories are only computed immediately before the motion is executed.

The STS motion has been analyzed in the biomechanics, optimization, and robotics spaces. The movement speed in the elderly is found to be slower than the younger population, suggesting that a stable STS does not solely rely on leg muscle strength, but also the ability to maintain stability (Yamada and Demura, 2009). Optimal control was utilized to determine the best way possible for external forces to support an elderly person doing STS (Mombaur and Ho Hoang, 2017) and the optimal trajectory for the REEM-C humanoid robot to perform unassisted STS (Aller et al., 2022). Recently, an impedance modulation control that accounts for balance reinforcement and impedance compensation is created to assist STS motion of a person wearing the Angeleg exoskeleton (Huo et al., 2022).

Although STS has been investigated, it has not been explored in detail for lower-limb exoskeletons using optimal control and motion capture. Particularly, the whole-body biomechanics, including arm movements, of a crutch-less STS motion is not yet addressed. Modifying an existing lower-limb robotic exoskeleton to be safe and suitable for the frailer geriatric population requires extensive research and analyses. This paper is a computational investigation on the biomechanics of performing STS without crutches with a lower-limb robotic exoskeleton. It first describes a motion capture experiment conducted to reveal the kinematics and forces of performing STS with crutches with the TWIN exoskeleton's default trajectory. Next, it describes the kinematics of performing STS without crutches with a human-driven trajectory when wearing the TWIN unactuated. This paper then proceeds into three STS simulation scenarios performed with optimal control: (1) analyzing the crutch-less human-driven trajectory collected from motion capture, (2) generating a crutch-less trajectory involving a model of an elderly woman wearing TWIN, and (3) generating a crutch-less trajectory involving a model of only the elderly woman. The minimum exoskeleton motor torque needed to provide full assistance are identified, results are compared, and future improvements on expanding these findings are discussed.

## 2 Methods

This section first describes the motion capture experiments, then proceeds into the human models utilized, the modeling of STS, and the optimal control formulation for motion analysis and motion synthesis.

### 2.1 Motion capture of STS motions with and without crutches

**Figure 1:**
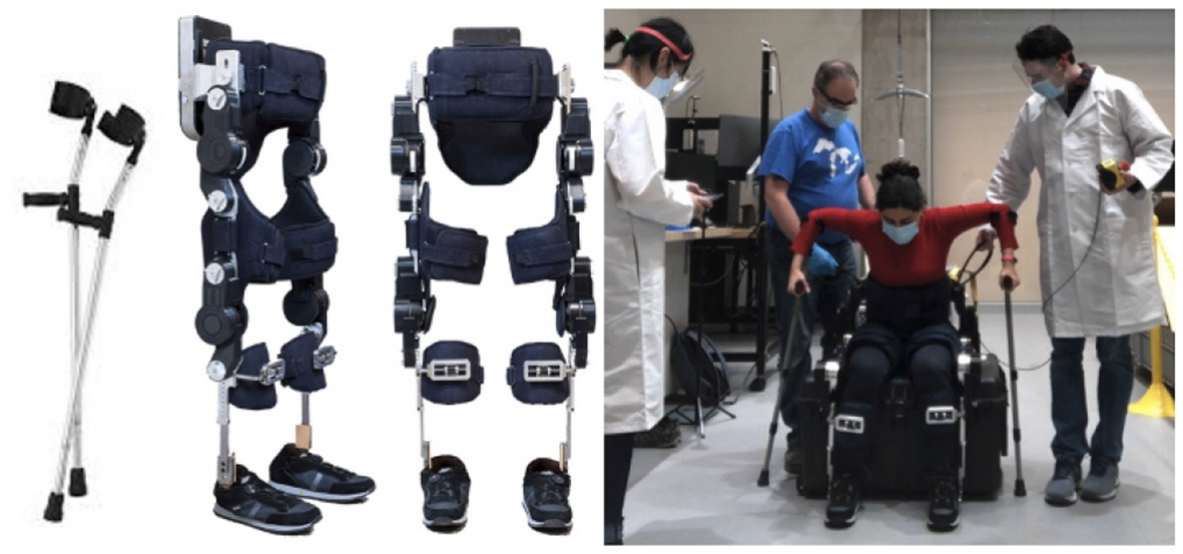
TWIN exoskeleton made by IIT heavily relies on crutches for safety and to push off for STS.

TWIN is a 25-kg lower-limb exoskeleton used in this study. Shown in Figure 1, it has four active DOFs in the hips and knees, and is made for people with paraplegia (Vassallo et al., 2020). The device is controlled by a tablet connected via Bluetooth, and the tablet is operated by another person. TWIN has two walking modes: each step is either manually triggered by pressing a button in Manual Walk Mode, or each step is initiated by the wearer based on the exoskeleton trunk's forward incline in Automatic Walk Mode. TWIN can also sit and stand, such that the kinematics of the sitting trajectory is an opposite of the standing trajectory. To walk, sit, and stand with TWIN, one must use crutches for safety and stability. Particularly for sit and stand, the person must properly push up (for standing) or support (for sitting) from behind, otherwise they can fall backwards.

Two motion capture scenarios, which are part of a larger dataset, are collected in this study:

1. Person moving with exoskeleton's default STS trajectory with crutches, with seat height at ~100% of the user's knee height.
2. User-driven trajectory with passive exoskeleton (i.e., motors disengaged) without crutches, with seat height at ~120% of the user's knee height.

The first scenario aids in understanding the dynamics of the exoskeleton's default STS trajectory, which can only be activated at a specific seat height due to predefined initial joint angles. The second scenario does not demonstrate the intended use of the device; it simply shows that it is possible to stably do crutch-less STS with TWIN for feasibility reasons. A higher seat height is chosen for the second scenario because we assume it is easier to perform the motion at a higher seat height.

One 25-year-old female participant experienced in moving with TWIN was recruited for a pilot study. This is because the second scenario is just for showing the feasibility of crutch-less STS and not a lot of people can do it. Kinematics are recorded with a 12-camera Vicon Vantage motion capture system. A modified version of the Istituto Ortopedico Rizzoli (IOR) marker set is used (Leardini et al., 2007). To obtain more accurate translation and position values, the Vicon skeleton model (VSK) is modified on Procalc. Forces exerted at the crutches/feet are captured with Bertec force plates. The human data collected from this study has received ethics clearance from the ethics board of the University of Waterloo.

### 2.2 Optimization-based investigation

**Figure 2:**
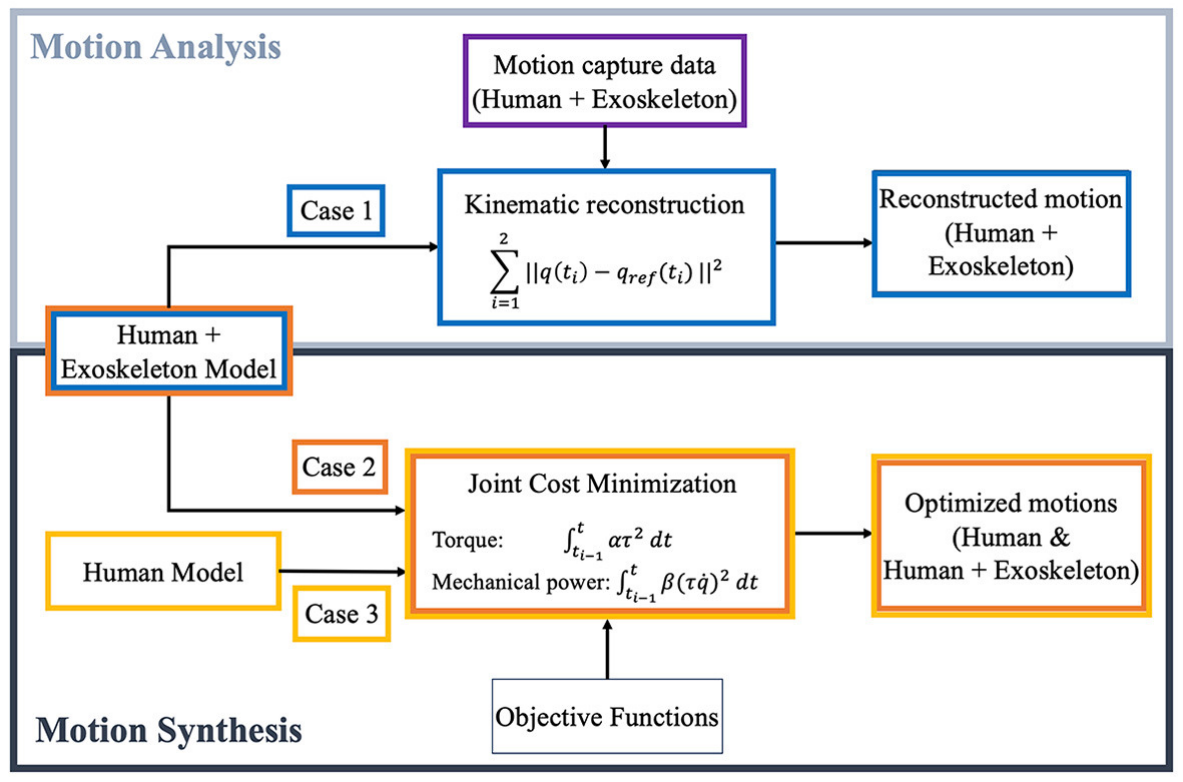
The optimal control approaches taken to evaluate and compare the dynamics of sit-to-stand: **(top)** perform motion analysis on motion capture recording (human-driven crutch-less STS with exoskeleton unactuated) by minimizing distance between simulation and reference position trajectories, and **(bottom)** generate optimal STS trajectories using human models with and without exoskeleton.

Besides performing motion capture experiments, STS is also investigated using numerical optimization techniques. The three simulation scenarios are described as follows and summarized in Figure 2.

1. Motion analysis on the motion capture data of the person doing STS with passive TWIN without crutches.
2. Motion synthesis of an elderly woman with TWIN doing STS without crutches.
3. Motion synthesis of an elderly woman without TWIN doing STS without crutches.

In the motion synthesis scenario with TWIN, the resulting torques are assumed to be an ideal combination of torques exerted by the elderly woman and device. Therefore, the purpose of including the motion synthesis scenario without TWIN is for determining whether the elderly woman's torque contribution is feasible.

#### 2.2.1 Dynamic models

**Figure 3:**
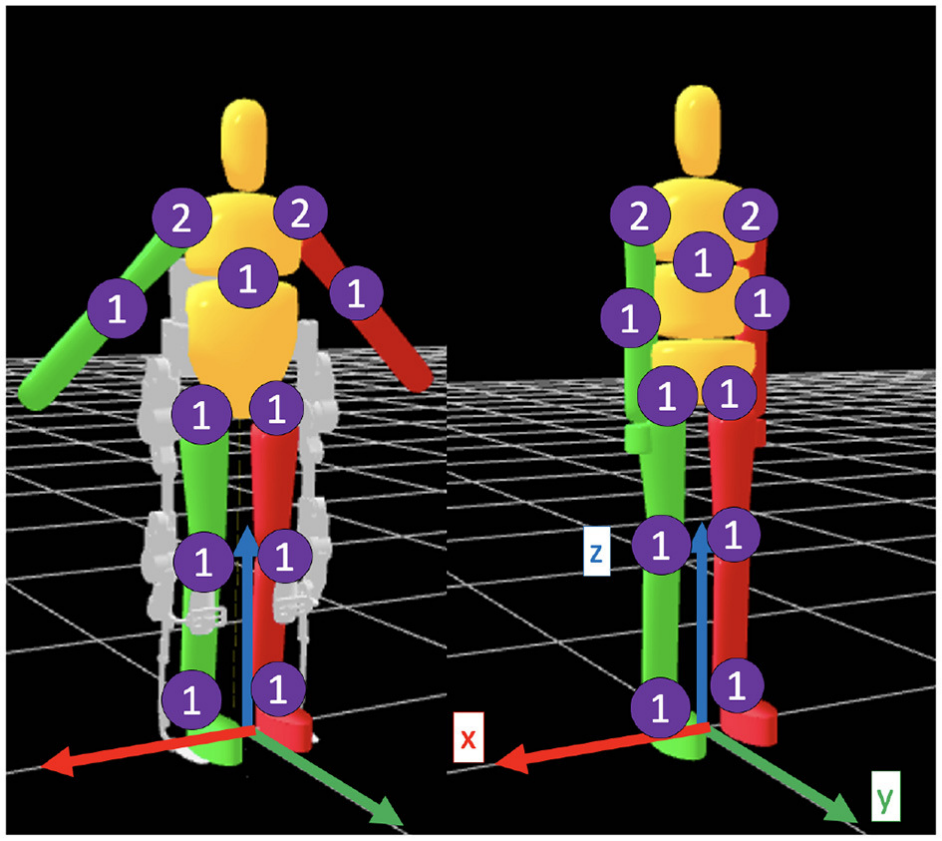
Visualization of the dynamic models with the exoskeleton **(left)** and without **(right)**. The numbers denote DOF in each joint.

Two dynamic models, visualized in Figure 3, are created to describe an elderly woman with and without the TWIN exoskeleton. The human model parameters are based on adjusted de Leva anthropometric values for a 50th percentile elderly woman (Ho Hoang and Mombaur, 2015), and the exoskeleton model parameters are scaled to the human's. Lower-limb symmetry is assumed along the sagittal plane since the STS motion is symmetrical in nature. TWIN's trunk brace limits movement in the lumbo-sacral joint, so the human-TWIN model is reduced to 10 physical segments: head, upper trunk, midtrunk-pelvis (lumped), thighs, shanks, feet, upper arms, and forearms. The ergonomics of optimal STS trajectories are not of interest, so the neck joint is fixed. Created as a bioMod file, the lumped model has 10 torque actuators: the xiphisternal joint, hips, knees, ankles, shoulders (frontal and sagittal), and elbows. The human-only model is constructed in a similar fashion and excludes TWIN's mechanical properties.

#### 2.2.2 Sit-to-stand model

STS is divided into sitting and lifting phases. Feet and seat contact constraints are present in the sitting phase, whereas only feet contact constraints are present in the lifting phase. They can be described by the set of differential algebraic equations (DAE) shown in Equation 1, though note that the set changes for each phase.


(1a) $$\begin{array}{l} \dot{q}=v \end{array}$$

(1b) $$\begin{array}{l} \dot{v}=a \end{array}$$

(1c) $$\begin{array}{l} \left(\begin{array}{cc} M & G^{T}\\ G & 0 \end{array}\right)\left(\begin{array}{c} a\\ \lambda \end{array}\right)=\left(\begin{array}{c} -N+F\\ -\gamma \end{array}\right) \end{array}$$

(1d) $$\begin{array}{l} g_{pos}=g\left(q\left(t\right),p\right)=0 \end{array}$$

(1e) $$\begin{array}{l} g_{vel}=G\left(q\left(t\right),p\right)\cdot \dot{q}=0 \end{array}$$

**Figure 4:**
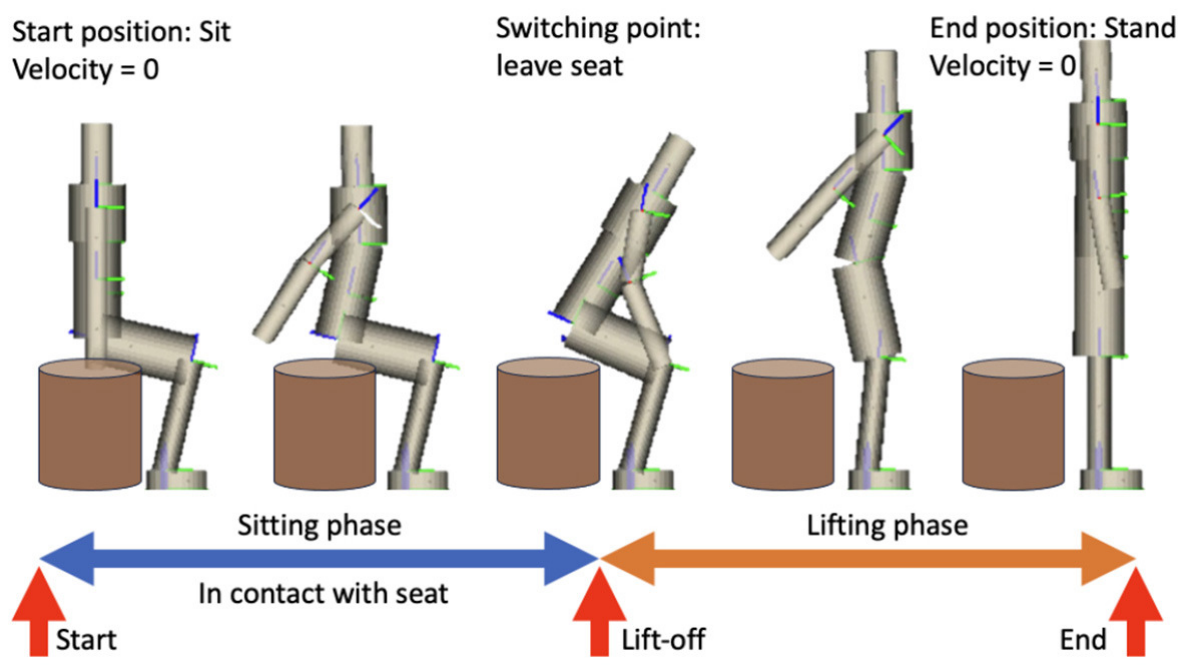
Visualization of STS and the phases involved. Initial, switching, and final conditions are indicated.

In this context, *q*, *v*, and *a* are positions, velocities, and accelerations respectively. *M* is the mass or inertia matrix, *N* is the vector of nonlinear effects, and *F* is the vector containing all external forces (gravity, drag, joint torques, etc.). The Jacobian matrix *G* of the position constraints is *G* = ∂*g*/∂*q*. The corresponding Hessian, γ, can be expressed as $\gamma =\left(\left(\partial G/\partial q\right)\dot{q}\right)\dot{q}$. The Lagrange multipliers, λ, are equivalent to the contact forces from the position constraints declared in Equation 1d. The switch from sitting to lifting phase occurs when the vertical contact force in the seat becomes zero, i.e., when the corresponding Lagrange multipliers become zero. Phase transitions are continuous, and unilateral constraints are present to ensure the forces exerted do not pull the lumped model into the ground nor the seat. The motion is summarized in Figure 4.

#### 2.2.3 Optimal control

Optimal control is an optimization-based approach to determine the control and state trajectories over a time period, such that an objective function related to said controls and states are minimized (Siciliano et al., 2009). Since it is able to determine the required joint actuation of a recorded motion (Koch and Mombaur, 2015) and also generate an optimal trajectory (Hu and Mombaur, 2017; Mombaur and Ho Hoang, 2017; Aller et al., 2022), this method is chosen for the computational STS investigation presented. The dynamic process of interest is a person, without using crutches, performing STS with and without the TWIN exoskeleton. The states, *x*, are position, velocity, and joint torque ${\left[q,\dot{q},\tau \right]}^{T}$ and the controls are the first derivative of the joint torques $\dot{\tau }$. To solve the optimal control problems (OCPs), the direct multiple-shooting method is used for its capability of yielding very accurate results for multi-phase problems. One can use the Rigid Body Dynamics Library (RBDL; Felis, 2017) with MUSCOD-II (Leineweber et al., 2003) to solve multi-phase OCPs. Another available library that can solve multi-phase OCPs with the direct multiple-shooting method is bioptim (Michaud et al., 2023). It is open-source and utilizes biorbd (Michaud and Begon, 2021) and CasADi (Andersson et al., 2019) to perform optimization problems through a Python interface. Users can choose between IPOPT (Waechter and Biegler, 2006) and acados (Verschueren et al., 2019) to solve OCPs. In this paper, bioptim with IPOPT is used and the results are visualized on bioviz. ^3^

Motion analysis is performed via least-squares position tracking and utilizes the dynamic model of the elderly woman wearing TWIN. *q* represents the joint angular positions generated by the OCP. *q*_*ref*_ represents the joint angular positions from the reference data, which is obtained from the motion capture recording (human-driven crutch-less STS with exoskeleton unactuated). Another small regularization term, ϕ(·), is added to maintain smoother controls. The full OCP formulation for motion analysis is expressed in Equation 2. The young adult subject and geriatric dynamic model have different segment lengths, so floating base translation values are scaled accordingly.


(2a) $$\begin{array}{l} \underset{x\left(\cdot \right),u\left(\cdot \right)}{\min}\;\overset{2}{\underset{i=1}{\sum }}\left(\|q\left(t_{i}\right)-q_{ref}\left(t_{i}\right){\|}^{2}+\int_{t_{i-1}}^{t_{i}}\phi \left(\cdot \right)dt\right) \end{array}$$

(2b) $$\begin{array}{l} \text{s.t. }ẋ\left(t\right)=f_{i}\left(t,x\left(t\right),u\left(t\right),p\right)\;\text{for }t\in T \end{array}$$

(2c) $$\begin{array}{l} r_{eq}\left(x\left(0\right),...,x\left(T\right),p\right)=0 \end{array}$$

(2d) $$\begin{array}{l} r_{ineq}\left(x\left(0\right),...,x\left(T\right),p\right)\geq 0 \end{array}$$

(2e) $$\begin{array}{l} g_{i}\left(t,x\left(t\right),u\left(t\right),p\right)\geq 0\;\text{for }t\in T \end{array}$$

(2f) $$\begin{array}{l} T={\left[t_{1},t_{2}\right]}^{T} \end{array}$$

*i* is the index representing each of the two phases (sitting and standing). *T* is a vector of the phase durations. *x* and *p* are the states and parameters of the OCP respectively. Equation 2b is the system dynamics and the DAE that describes the STS motion. Equations 2c, 2d are the equality and inequality boundary constraints at different points throughout the two phases. Equation 2e is the box constraints on the system's states: ${\left[q,\dot{q},\tau \right]}^{T}$. The bounds for state variables are declared such that they do not hinder the position tracking problem, and no box constraints are added for the controls.

The objective function in motion synthesis minimizes joint torques squared and joint mechanical power squared. Mombaur and Ho Hoang stated that the former term is crucial in optimizing dynamic motions, and the latter term makes the resulting motion less dynamic and therefore more appropriate for older adults (Mombaur and Ho Hoang, 2017). The full formulation is described in Equation 3. To compare how the generated motion and torques differ with and without TWIN, motion synthesis is performed twice with the corresponding dynamic models.


(3a) $$\begin{array}{l} \underset{x\left(\cdot \right),u\left(\cdot \right),T}{\min}\;\overset{2}{\underset{i=1}{\sum }}\int_{t_{i-1}}^{t_{i}}\left(\overset{n_{act}}{\underset{j=1}{\sum }}\alpha \tau_{j}^{2}+\beta {\left(\tau_{j}{\dot{q}}_{j}\right)}^{2}\right)dt \end{array}$$

(3b) $$\begin{array}{l} \text{s.t. }ẋ\left(t\right)=f_{i}\left(t,x\left(t\right),u\left(t\right),p\right)\text{ for }t\in T \end{array}$$

(3c) $$\begin{array}{l} r_{eq}\left(x\left(0\right),...,x\left(T\right),p\right)=0 \end{array}$$

(3d) $$\begin{array}{l} r_{ineq}\left(x\left(0\right),...,x\left(T\right),p\right)\geq 0 \end{array}$$

(3e) $$\begin{array}{l} g_{i}\left(t,x\left(t\right),u\left(t\right),p\right)\geq 0\;\text{for }t\in T \end{array}$$

(3f) $$\begin{array}{l} T={\left[t_{1},t_{2}\right]}^{T} \end{array}$$

α and β are the respective weights for the objective function terms joint torque squared and joint mechanical power squared. *n*_*act*_ denotes the number of actuated DOFs in the model with *j* as its index. τ_*j*_ and $\dot{q_{j}}$ refer to the joint torque and joint velocity of each actuated DOF. Equations 3b–3d are similar to Equations 2b–2d since they represent the system dynamics of STS (DAE from Equation 1), equality boundary constraints, and inequality boundary constraints respectively. The bounds for states and controls are the same for OCP synthesis with and without TWIN, except for the ankle torque, which are determined such that the unilateral constraints between the feet and ground are reinforced. Since the presence of the exoskeleton changes the maximum torque the bottom of the feet can experience before the toe/heel lifts off the ground (aka disobey the ground contact constraints), the ankle bounds in OCP synthesis without TWIN is smaller.

## 3 Optimization results and comparison with motion capture

**Figure 5:**
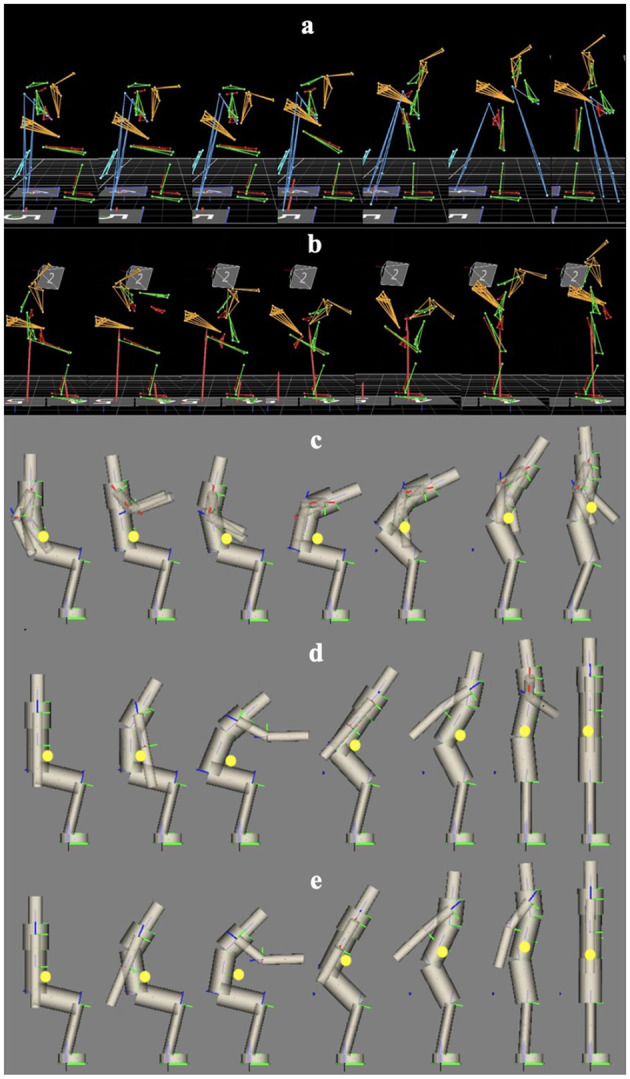
**(a)** Motion capture frames of actuated exoskeleton with crutch support. **(b)** Motion capture frames of passive exoskeleton without crutch support. **(c)** Simulation frames of OCP analysis with exoskeleton without crutch support. **(d)** Simulation frames of OCP synthesis with exoskeleton without crutch support. **(e)** Simulation frames of OCP synthesis without exoskeleton without crutch support. Yellow dots represent the model's total CoM. See Supplementary material for the full videos.

The research presented in this paper first investigates the default exoskeleton STS dynamics with crutches, then combines motion capture and optimal control methods to evaluate and compare the crutch-less STS dynamics with the exoskeleton. The underlying joint torques of the human-driven trajectory from motion capture data and the optimal crutch-less STS trajectories are successfully obtained. It is once again emphasized that the crutch-less scenario in the motion capture experiment is performed to only show the feasibility and possibility of doing crutch-less STS with TWIN, and the data act as a reference and preliminary step towards determining an optimal trajectory. The motion capture and simulation frames can be found in Figure 5, and videos of these motions can be found in the Supplementary Material. The yellow dots in Figure 5 reflect the model's total center of mass (CoM), which will be discussed later.

### 3.1 Motion capture findings

**Figure 6:**
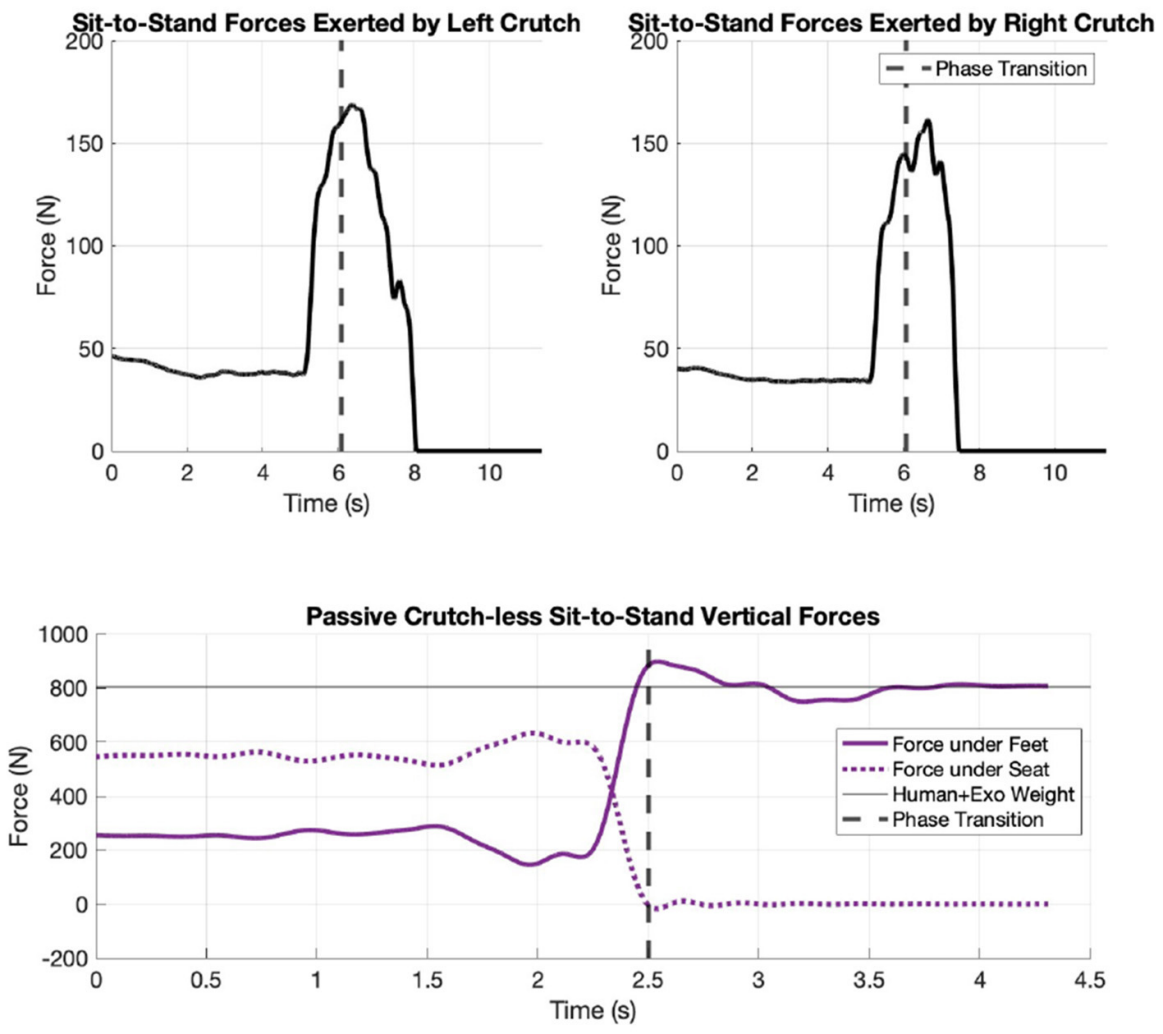
Force plate data from motion capture trials: crutch forces from default exoskeleton trajectory **(top two in black)** and feet and seat forces from user-driven crutch-less scenario **(bottom in purple)**.

**Figure 7:**
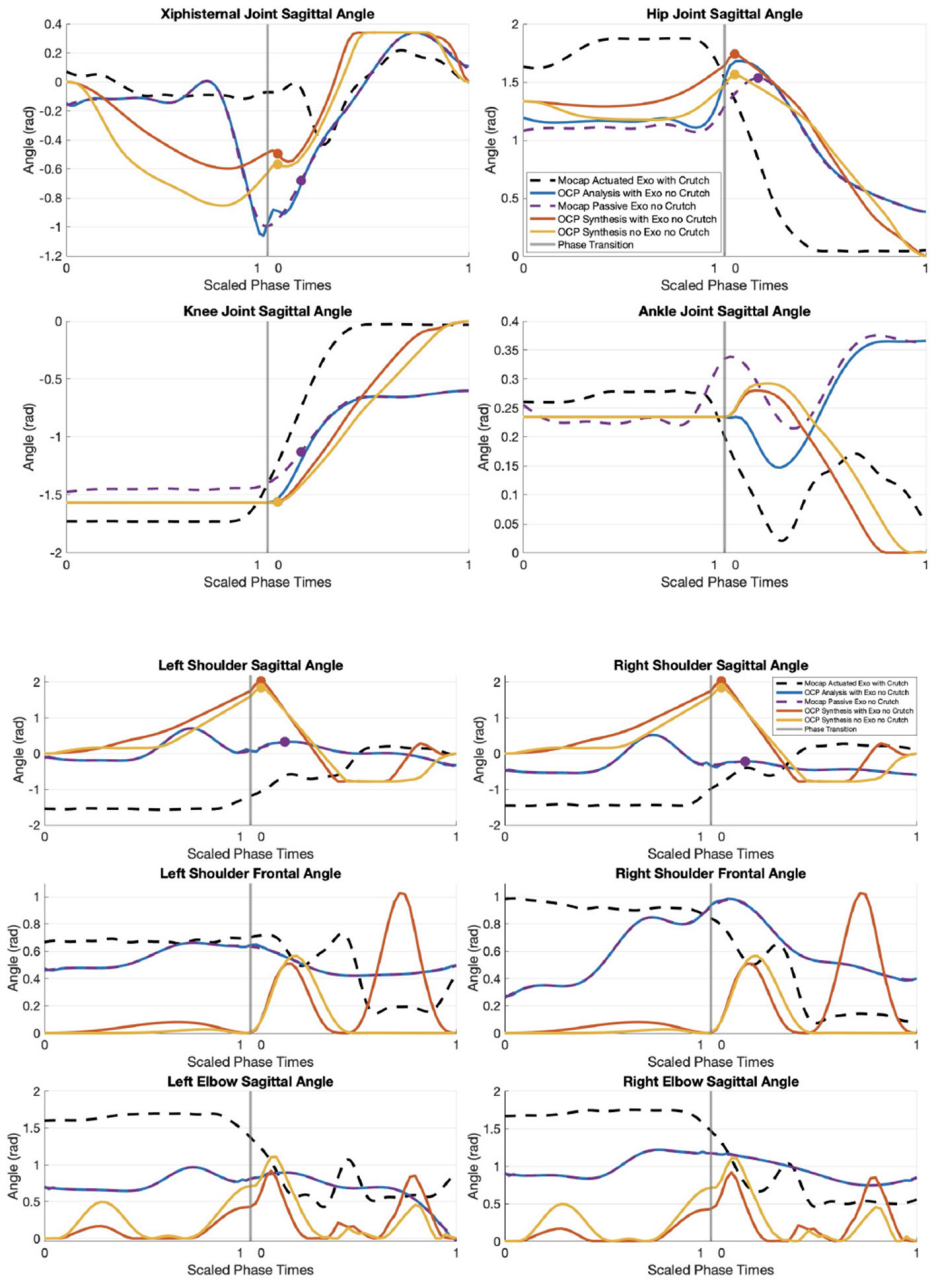
Position plots from motion capture and OCP results. Dots in xiphisternal, hip, knee, and shoulder sagittal angle plots represent the moment of maximum hip flexion.

The first scenario involving the default exoskeleton trajectory lasts 11.36 s, which includes 2 s to bend the trunk to prepare for STS, 2 s to pause between trunk bend and standing up, and 4 s to stand up from sitting. The total mass of the human, exoskeleton, and crutches is 83.1 kg and the peak combined ground reaction force in the crutches is measured to be 330.3 N (see Figure 6). Using Equation 4 and estimating the CoM acceleration with the floating base origin acceleration, the force at feet is estimated to be 484.9N. The hip and knee angles describe the default motor trajectories, whereas the xiphisternal and ankle joints represent the user's behavior. The shoulder angle plots depict that the crutches are first placed behind the body to push off during lifting phase, and later placed forward for balance once standing. The position plots can be found in Figure 7.


(4) $$\begin{array}{l} F_{crutches}+F_{feet}=M_{total}*\left(a_{COM}+g\right) \end{array}$$

Force plate results reveal that a peak maximum ground reaction force of 330 N is required in the crutches. The participant also made a remark on shoulder discomfort, yet the ready position is required for a proper push-off for the lifting phase. The kinematics results show an initial shoulder extension of 1.4 rad and the entire STS duration lasts 11.36 s.

The second scenario lasts 4.3 s. The total mass of the human and exoskeleton is 82 kg and the feet forces reach a peak value of 898.0 N. This indicates that the user must accelerate upwards to stand up from sitting. The kinematics data show that the person first swings their arms and bends their trunk forward before standing up. The shoulder range of motion (ROM) is less than the first scenario because the user does not have to bring the crutches from the back to the front of the body. The force plots can be found in Figure 6, and the joint position plots can be found in Figure 7. The position plots and the calculated underlying joint torques from the crutch-less scenario will be analyzed in more detail later.

### 3.2 Joint angular position from OCP results

Figure 7 illustrates the angular positions in the lower and upper body joints in all motion capture and OCP scenarios.

In OCP analysis, the optimizer is able to closely track upper body joints and there are minor tracking differences in the lower limb joints. A maximum offset of 0.27, 0.19, and 0.10 rad are observed in the hip, knee, and ankle joints respectively.

Comparing the hip joint angles from OCP synthesis results, the scenarios OCP analysis (0.57 rad) and motion capture passive TWIN without crutches (0.46 rad) exert the highest upper body forward bend. OCP synthesis with TWIN shares a similar forward bend angle of 0.45 rad, and OCP synthesis without TWIN exerts 0.39 rad of forward bend.

The final position for the hip, knee, and ankle joints are 0 rad for the OCP synthesis solutions, which means the model is standing straight. Meanwhile, the values in OCP analysis are 0.379, –0.602, and 0.363 rad respectively.

When standing up, the OCP synthesis results share a similar pattern of flexing the upper trunk forward, but the OCP analysis slightly arches the upper trunk back before flexing forward. The motion capture result with passive TWIN without crutches flexes up to 1rad, whereas the OCP analysis flexes up to 1.06 rad. The OCP synthesis with TWIN flexes up to 0.60 rad, though the OCP synthesis without TWIN has a larger flexion of 0.85 rad.

Both OCP synthesis solutions encourage the elderly woman model to swing her arms forward in the sitting phase, and swing the arms back while abducting the shoulders during lift-off. Towards the end of the lifting phase, OCP synthesis with TWIN recommends the elderly woman to abduct the shoulders when bringing the arms to the front, though OCP synthesis without TWIN does not show shoulder abduction or adduction at this stage.

### 3.3 Timing comparison between motion capture and OCP solutions

The duration of each phase in the OCP analysis scenario must be aligned with the user-driven trajectory motion capture data because of the problem formulation. The OCP synthesis scenarios left time as a free variable ranging between 0.5 and 2 s per phase, and the formulation does not directly minimize time. The sitting and standing duration are expressed in Table 1.

**Table 1:** Lifting and sitting phase times for motion capture trials and OCP results.

	**Time (s)**	
	**Sitting phase**	**Lifting phase**
Motion capture actuated TWIN with crutches	6.08	5.28
Motion capture user-driven trajectory passive TWIN without crutches	2.50	1.80
OCP Analysis with TWIN without crutches	2.50	1.80
OCP Synthesis with TWIN without crutches	0.70	2.00
OCP Synthesis without TWIN without crutches	0.85	1.67

In the passive TWIN crutch-less motion capture data, OCP analysis, and OCP synthesis with and without TWIN, the xiphisternal joint consistently reaches maximum flexion before the hips. At the moment of maximum hip flexion in the passive TWIN crutch-less motion capture data, there is a second peak in shoulder flexion and the knee is already extending (see purple dots in Figure 7). In the OCP synthesis results with and without TWIN, maximum shoulder flexion occurs at the same instance of maximum hip flexion and immediately before knee extension (see yellow and orange dots in Figure 7).

### 3.4 Center of mass

**Figure 8:**
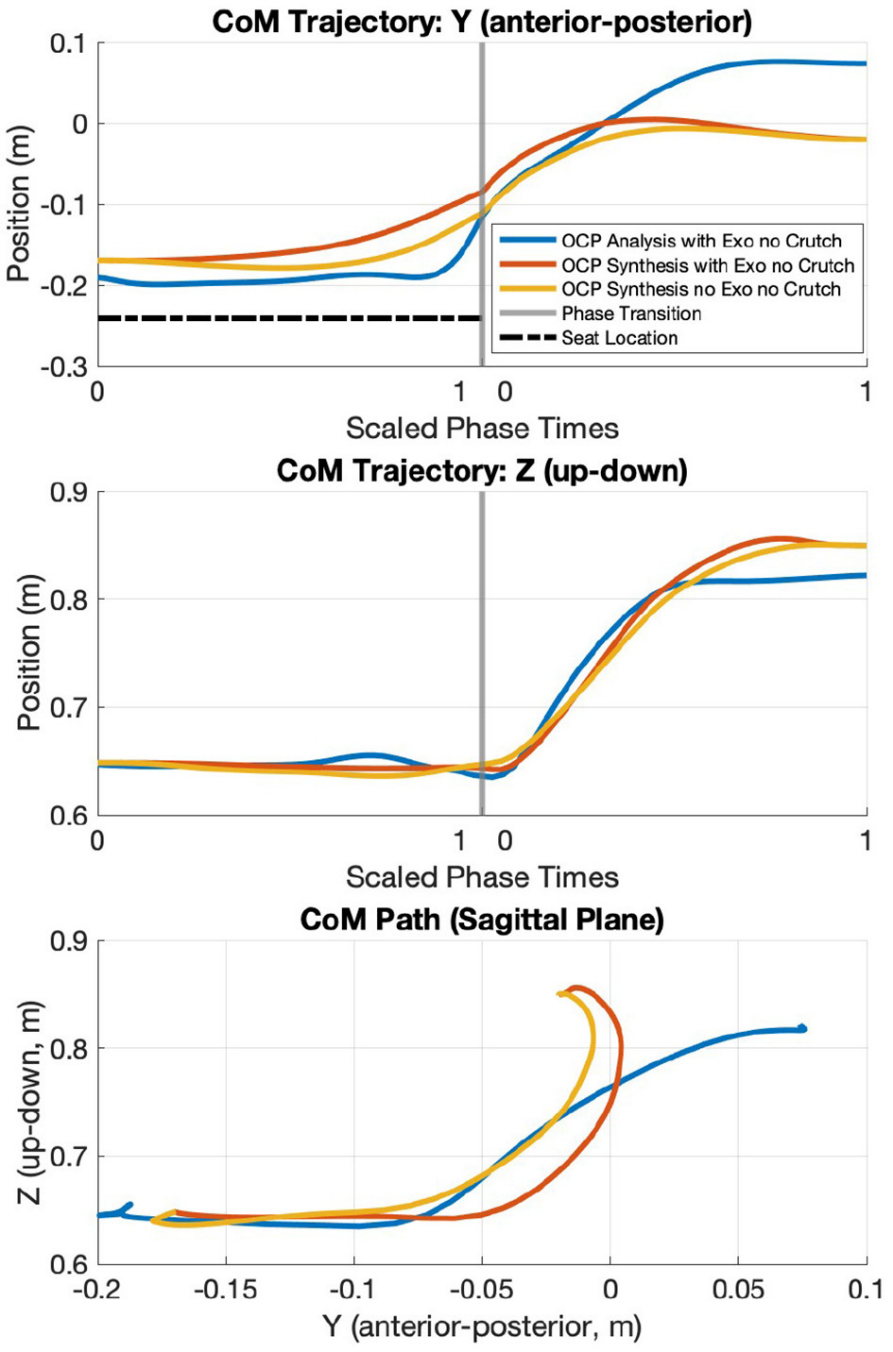
CoM trajectories from OCP results (motion analysis, motion synthesis with TWIN, and motion synthesis without TWIN). +*Y* is the anterior direction, and *Z* is the vertical distance from the ground.

The CoM trajectory is computed for all OCP scenarios using biorbd. Since 2D dynamic models with 3D shoulder joints are used, the overall CoM trajectories for the medial-lateral direction are zero and hence omitted. In motion analysis, the CoM trajectory begins at a more posterior position and ends at a more anterior position than the synthesis cases. During lifting phase, a slight decrease in the *Y*-axis of the CoM trajectory is observed in both motion synthesis cases, which corresponds to the CoM paths curving out slightly anteriorly (see Figure 8).

### 3.5 Joint torque from OCP results

**Figure 9:**
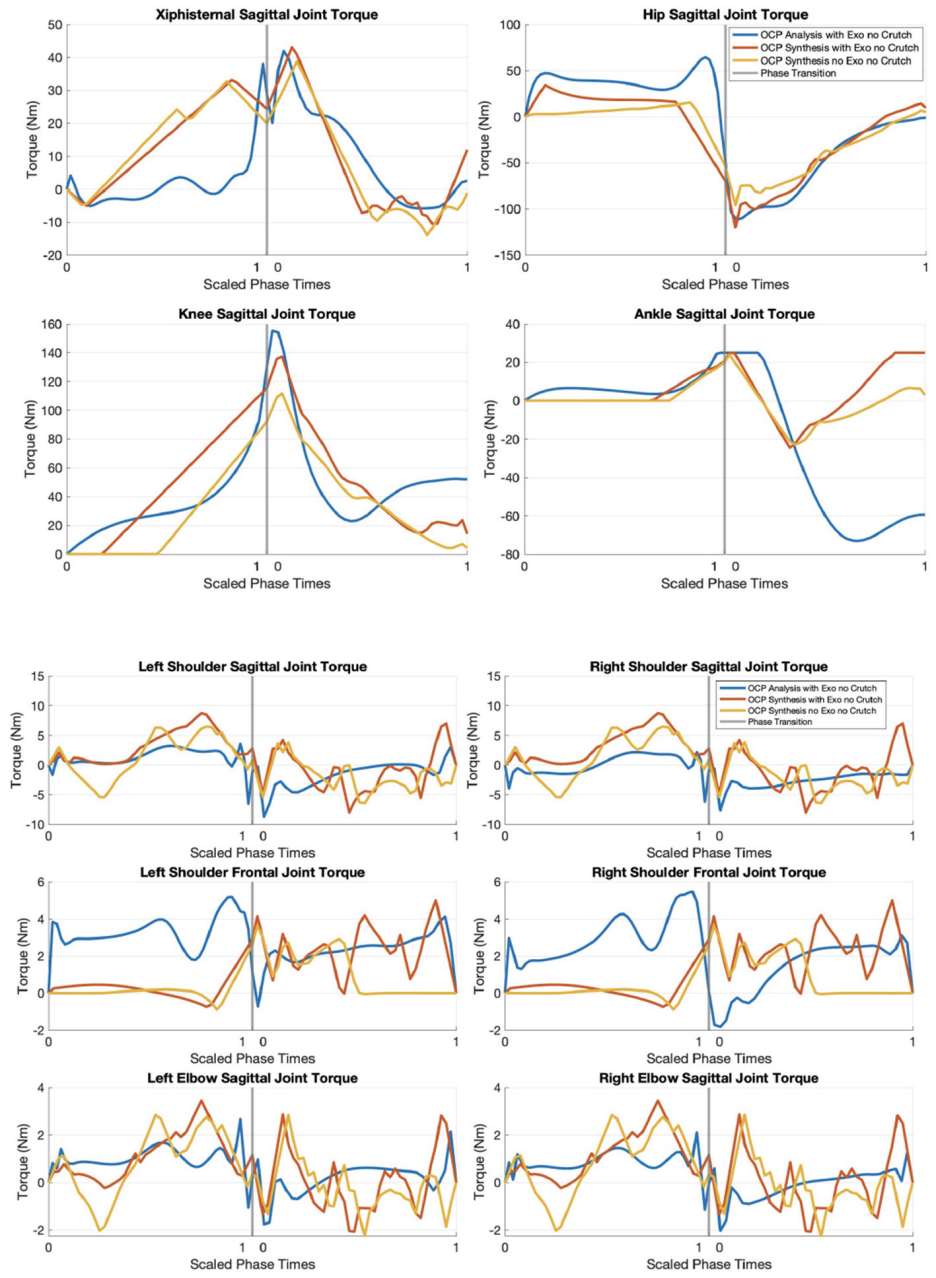
Torque plots from OCP results (motion analysis, motion synthesis with TWIN, and motion synthesis without TWIN).

Figure 9 shows the joint torques from OCP analysis, OCP synthesis with TWIN, and OCP synthesis without TWIN. Recall that left and right lower limbs are lumped due to STS' symmetrical nature, so the torque values for these joints are the sum of both sides. The torques calculated from the motion capture data are based on motions performed with a passive exoskeleton, meaning the motors are disengaged, and the bioMod files do not include any information about motor friction. Therefore, the torques reported in this paper are technically net torques. In the OCP analysis, the values are the torques exerted by the human participant, whereas the values in OCP synthesis with TWIN are the total ideal torque combination of human and exoskeleton.

The xiphisternal joint exerts the highest torque in the OCP synthesis scenario with TWIN (43.12 Nm) and the least in the OCP synthesis scenario without TWIN (38.77 Nm). The OCP analysis scenario exerts less hip joint torque than OCP synthesis with TWIN, but applies the most knee and ankle torques.

The highest peak frontal shoulder joint torque is observed in OCP analysis because the arms are consistently abducted to avoid collision with the exoskeleton structure (see angle in Figure 7). The torque trajectories from the two OCP synthesis solutions seem jerky in the upper limbs, but note that the maximum range of the torque values are within ± 9 Nm.

Combining left and right, 120.03 Nm in the hips and 137.26 Nm in the knees are required to perform STS in OCP synthesis with TWIN. In reality, the device's existing hip and knee motors can exert a total of 112 and 88 Nm respectively (Vassallo et al., 2020), meaning it can only provide partial STS assistance. According to the results from OCP synthesis without TWIN, the elderly woman would be able to exert a maximum absolute torque of 95.79 and 111.79 Nm in the hips and knees respectively. Assuming an ideal combination from the human and exoskeleton, the user would need to contribute a total of 8.03 Nm in the hips and 49.26 Nm in the knees. These values are summarized in Table 2.

**Table 2:** OCP synthesis results: maximum absolute torque comparison between with and without TWIN, and determining torque contributions assuming ideal sharing between older adult and exoskeleton.

	**Without TWIN (Nm)**	**With TWIN (Nm)**		
**Joints**	**Human**	**Human + Exo**	**Exo**	**Human**
Hips	95.79	120.03	112	8.03
Knees	111.79	137.26	88	49.26

## 4 Discussion

### 4.1 Joint angular position

In OCP analysis, the tracking offset observed in the hip, knee, and ankle joints could stem from different reasons. The offset could be influenced by the very small regularization term ϕ(·) in the objective function, which prevents the joint positions from tracking the reference data perfectly. Another reason could be the slight difference in model definition between the VSK and bioMod. The segments declared in the VSK heavily depend on the motion capture marker layout. Given the presence of TWIN, some markers in the original IOR marker set must be relocated to accommodate the device and avoid marker occlusion. Although one can redefine the variables on Procalc to reduce model discrepancy between the VSK and bioMod, there will always be some error, thus introducing an offset in the tracking results. A third reason could be related to the motion capture data. When recording the user-driven STS scenario involving a passive TWIN without crutches, it is possible that the person did not begin the motion at exactly zero velocity and zero torque. Figure 4 emphasizes that STS is to be modeled with zero velocity at the start, so the offset could be a result of this constraint contradicting the measurements.

Regarding the upper body forward bend in the lifting phase, OCP analysis' minor tracking error in the hip joint has led to a 0.11 rad increase compared to the motion capture data of passive TWIN without crutches. That said, it is reasonable to see a larger bend angle when an exoskeleton is worn, since more effort is required to move with the additional mass.

The hip, knee, and ankle final positions from OCP analysis in Figure 7 do not reflect that the person in the motion capture experiment is not fully standing up. This is caused by a fitting error between the younger adult and TWIN, such that the exoskeleton's lower limbs are longer than the human's. Although the exoskeleton is customized to the wearer's size, fine adjustments in the modules cannot be made to allow for a perfect fit. Therefore, even when the person's legs are vertical, the exoskeleton segments remain bent. With the motion capture markers placed on the exoskeleton segments, the fitting error is propagated into the position and torque profiles of these joints.

Since TWIN is passive and all the actuation originates from the human's joint in the crutch-less motion capture scenario, it is reasonable to observe the largest flexion in the xiphisternal joint since the person is generating as much forward angular momentum as possible to stand up without falling. On the contrary, OCP synthesis with TWIN has the least flexion in the xiphisternal joint, so further investigation is recommended.

The arm-swing results suggest that the sagittal and frontal arm movements could aid in generating angular momentum and maintaining stability to stand up without crutches. Note that the average shoulder frontal joint angles are larger in OCP analysis, since the person is preventing the arm from swinging into the exoskeleton's structure in real life, yet this is not reflected in the OCP synthesis simulations. It is recommended to further investigate the effects of these arm motions on a stable and successful crutch-less STS motion.

### 4.2 Timing

When standing up without crutches with or without the exoskeleton, the timing coordination of maximum xiphisternal flexion first then maximum hip and shoulder flexion may suggest that this sequence is important for generating angular momentum to stand up without falling backwards. Knee extension begins at maximum hip and shoulder flexion in both OCP synthesis cases, but the start of knee extension and maximum shoulder flexion have already occurred before maximum hip flexion is reached in OCP analysis. It could be that the subject in the motion capture experiment has to overcome motor friction when moving, yet the bioMod model with TWIN does not include such information, hence the earlier occurrence of knee extension and shoulder flexion. Although keeping the amount of motor friction consistent across scenarios could possibly confirm the proper coordination, this is not possible given the circumstances of this study. Nonetheless, a future recommendation is to investigate the multi-joint coordination presented in the OCP synthesis results with and without TWIN.

Without an exoskeleton, it is assumed that it would take less time to complete both phases due to less mass and inertia. OCP synthesis without TWIN lasts 0.33 s less than OCP synthesis with TWIN in the lifting phase, yet it takes 0.15 s longer than OCP synthesis with TWIN in the sitting phase. It is unknown why this occurs, so more investigation is needed to explore the influence of ankle torque bounds.

The cardiovascular system becomes more affected as a person ages, so any sudden change in blood flow can be more pronounced and cause dizziness in an older adult. To create a STS trajectory suitable for the geriatric population, suggestions include investigating more weight combinations between the objective function's torque and mechanical power terms, tuning the torque-derivative bounds to better reflect the joint capabilities of an older adult, and incorporating the change in blood flow of the geriatric cardiovascular system in the formulation.

### 4.3 Center of mass

The dynamic models used in the simulations are primarily 2D, so all OCP cases do not have any CoM deviation in the medial-lateral direction. It is observed that the CoM trajectory in motion analysis begins more posteriorly and ends more anteriorly than in motion synthesis cases. Recall that motion analysis tracks the joint angular positions from the motion capture data. One reason could be the weight of the TWIN exoskeleton trunk module causing the participant to lean more backward during the sitting phase, and she has to lean forward to prevent herself from falling backwards at the end of lifting phase. Meanwhile, the motion synthesis cases assume the lower-limb joint torques to be the total ideal torque combination of human and exoskeleton, hence the smaller anterior-posterior range. Another reason for different CoM behaviors could be the influence of the angle of shoulder extension and xiphisternal joint flexion, but a detailed analysis would be required to identify the amount of influence each joint movement has on the CoM based on the STS trajectories.

### 4.4 Joint torque

It is reasonable for OCP synthesis without TWIN to exert the least torque in the trunk and lower limbs, since the absence of an exoskeleton requires less effort from these joints to generate forward momentum to stand up from sitting. When comparing the OCP analysis and OCP synthesis with TWIN results, it seems that more hip torque leads to less knee and ankle torques required to stand up, but further verification is needed.

It is unsure how much torque frailer older adults could exert in the xiphisternal joint given the ROM of the optimal results and torque value. It is therefore recommended to further tune the physical parameters of this joint, so that the resulting motion can better represent this population.

The range of upper limb joint torques are within ± 9 Nm, but they seem jerkier than those in the xiphisternal and lower limb joints. Future recommendations for smoother plots include decreasing the torque-derivative bounds and tuning objective function term weights.

TWIN's current motors are unable to fully assist an elderly woman to do STS, since the hips and knees could only exert a total of 112 and 88 Nm respectively. For full assistance, the device would need stronger motors that can exert a minimum total torque of 121 Nm in the hips and 140 Nm in the knees. However, assuming the ideal scenario where the torques required to perform crutch-less STS are shared between the exoskeleton and human, partial assistance is possible because the remaining contribution needed from the user is feasible per the torques calculated in OCP synthesis without TWIN.

The joint torques reported in this paper are specific to the 25-kg TWIN, but these values can be applicable to other exoskeletons of similar mass distributions. The presented optimal control approaches, in fact, can be replicated on any exoskeleton to generate the required joint torques for performing STS, supposing the mass distributions of the exoskeleton of interest are available. After determining the target population, one would create an appropriate dynamic model that also incorporates the device's mass distributions, formulate the OCP as described in Section 2.2.3, then run the simulation.

## 5 Conclusion and outlook

Lower-limb exoskeletons show the potential to assist frailer older adults to perform STS, but none of the devices in the market are currently suitable for this population, and the mandatory use of crutches imposes health challenges to them. As well, one of our motion capture experiments confirm that the exoskeleton's default STS trajectory is nonintuitive and uncomfortable to perform with the crutches. The first step towards eliminating external stability aid when performing STS with an exoskeleton is to evaluate the dynamics involved with and without crutches. The motion capture data of user-driven trajectory with TWIN demonstrates the possibility of doing the motion without crutches. The optimal control solutions suggest that arm flexion/extension and abduction/adduction aid in performing a successful crutch-less STS. It is recommended to investigate deeper into the whole-body movement coordination presented in the OCP synthesis solutions, since it can possibly explain the coordination needed to successfully stand up with and without an exoskeleton from sitting without crutches. With a dynamic model involving a 50th percentile elderly woman, results show that TWIN's current motors are sufficient for partial assistance, but stronger motors are required for full assistance. To make the motion suitable for geriatric users, future recommendations include fine-tuning the objective function weights and accounting for the change in blood flow in geriatric cardiovascular system via modeling or as an objective function. The results presented are promising and are considered a foundation towards generating an appropriate crutch-less STS trajectory for older adults.

Not only can the optimal control approaches described be replicated on other exoskeletons, the works of this paper can also expand into multiple research avenues. With some adjustments to reflect the human height and lumped segment masses involved, one can implement the lower limb joint torque trajectories into existing exoskeletons in the form of partial or full assistance. In the former case, the user can choose the amount of assistance based on their capacity. The OCP synthesis approaches can also be performed with a 3D dynamic model, such that one can investigate how other 3D degrees-of-freedom behave to compensate for the purely-sagittal lower-limb movements. Other types of exoskeleton movements can also be generated using optimal control, including and not limited to flat-ground or sloped walking, perturbation recovery, and stair walking. To investigate the underlying effects on stability, it is recommended to analyze the generated arm motions and movement coordination between upper and lower limb joints.

## Data availability statement

The original contributions presented in the study are included in the article/Supplementary material, further inquiries can be directed to the corresponding author.

## Ethics statement

The studies involving humans were approved by Waterloo's Research Ethics Board. The studies were conducted in accordance with the local legislation and institutional requirements. The participants provided their written informed consent to participate in this study. Written informed consent was obtained from the individual(s) for the publication of any potentially identifiable images or data included in this article.

## Author contributions

JL: Methodology, Data curation, Formal analysis, Writing—original draft, Writing—review & editing, Investigation. KM: Methodology, Formal analysis, Funding acquisition, Supervision, Writing—review & editing.

## Footnotes

^1^ https://exoskeletonreport.com/product/easywalk/

^2^ https://exoskeletonreport.com/product/myosuit/

^3^ https://github.com/pyomeca/bioviz
